# Supplementary material for: Out-of-pocket costs for families and people living with cerebral palsy in Australia
Source: PLoS One. 2023 Jul 20;18(7):e0288865. doi: 10.1371/journal.pone.0288865 (PMC10358956; doi:10.1371/journal.pone.0288865)
Supplement: S2 Table — (DOCX) [file pone.0288865.s002.docx]

**S2 Table. Adult sociodemographic characteristics.**

| **Socio-demographic characteristic** | **Age 18+ n (%)** |
| --- | --- |
| Occupation of adult with CP |  |
| Education | 25 (25) |
| Full-Time Employment | 8 (8) |
| Part-Time Employment | 18 (18) |
| Supported Employment | 5 (5) |
| Unemployed, looking for employment | 6 (6) |
| Unemployed, not looking for employment | 10 (10) |
| Other Occupation  Missing/Unknown | 12 (12)  12 (12) |
| Living arrangements of adult with CP |  |
| With two parents | 43 (43) |
| With one parent | 11 (11) |
| With foster parent(s) | 1 (1) |
| With a partner/a partner and family | 13 (13) |
| Alone independently | 11 (11) |
| Alone with assistance | 4 (4) |
| With friends | 4 (4) |
| In a group home | 4 (4) |
| Other living arrangements  Missing/Unknown | 3 (3)  6 (6) |
| Highest level of education of adult with CP |  |
| Primary Education | 7 (7) |
| Secondary Education | 32 (32) |
| Vocational Training/Diploma | 16 (16) |
| Tertiary Education | 26 (26) |
| Other/None of the above  Missing/Unknown | 13 (13)  6 (6) |
| Before tax income of adult with CP (self-reporters only) |  |
| Less than $31*,*199 per year | 22 (54) |
| $31*,*200 to $51*,*999 per year | 6 (15) |
| $52*,*000 to $103*,*999 per year | 4 (10) |
| More than $104*,*000 per year | 4 (10) |
| Did not wish to disclose  Missing/Unknown | 4 (10)  1 (2) |
