# Supplementary material for: Out-of-pocket costs for families and people living with cerebral palsy in Australia
Source: PLoS One. 2023 Jul 20;18(7):e0288865. doi: 10.1371/journal.pone.0288865 (PMC10358956; doi:10.1371/journal.pone.0288865)
Supplement: S3 Table — (DOCX) [file pone.0288865.s003.docx]

**S3 Table. Percentage of participants who would like to spend more across cost types and GMFCS Level.**

| **Cost Type** |  | **Age 0-6 years**  **%** | **Age 7-17 years**  **%** | **Age 18 years +**  **%** |
| --- | --- | --- | --- | --- |
| Medical costs | GMFCS I-II | 22 | 45 | 39 |
|  | GMFCS III-V | 58 | 34 | 36 |
| Assistive tech | GMFCS I-II | 37 | 29 | 23 |
|  | GMFCS III-V | 58 | 61 | 42 |
| Home modifications | GMFCS I-II | 35 | 38 | 17 |
|  | GMFCS III-V | 67 | 66 | 75 |
| Occupation | GMFCS I-II | 15 | 40 | 18 |
|  | GMFCS III-V | 7 | 22 | 24 |
| Respite care | GMFCS I-II | 31 | 24 | 11 |
|  | GMFCS III-V | 42 | 35 | 24 |
| Holidays | GMFCS I-II | 50 | 61 | 58 |
|  | GMFCS III-V | 73 | 74 | 79 |
| Leisure | GMFCS I-II | 54 | 59 | 41 |
|  | GMFCS III-V | 59 | 56 | 72 |
