# Supplementary material for: Out-of-pocket costs for families and people living with cerebral palsy in Australia
Source: PLoS One. 2023 Jul 20;18(7):e0288865. doi: 10.1371/journal.pone.0288865 (PMC10358956; doi:10.1371/journal.pone.0288865)
Supplement: S1 Appendix — (PDF) [file pone.0288865.s004.pdf]

Financial costs for families and people living with cerebral palsy in Australia_Survey for a person living with CP

There are 12 short sections in this research survey.

Please read the information and questions attentively and click on

'save and return later' if you wish to pause.

Thank you for sharing your experience with us!

---

1. Information and consent

Project title

Financial costs for families and people living with cerebral palsy in Australia

Project summary

To determine out of pocket costs and measure financial distress for families and people living with cerebral palsy across age and severity.

Investigators

Claire Galea1,2, Dr Alison Pearce1, Dr Sarah McIntyre1,2, Isabelle Balde1,2, Sophie Marmont3, Fiona Garrity3, James Espie3

1University of Sydney, 2 Cerebral Palsy Alliance Research Institute, 3CP Quest Research Partner

Contact details

[redacted]

Consent

Completion of the survey will be considered as indication that you have read and understood this information and that you consent to your data being used for research purposes.

HREC

USYD HREC (2020/234 - date)

Time to complete survey

Between 30 to 90 minutes

With this research project we aim to estimate the financial costs of cerebral palsy (CP) and the level of financial stress for families each year in Australia.
We know that the impact of CP varies from person to person, and the support they require will vary too. We want to find out, from your perspective, the out of pocket costs associated with living with CP.
Results from this study will be written in a report that we will share with you and the broader CP Community. Findings may also be presented at conferences and published in academic journals.

We envisage the report created from this research will promote policy changes, as well as provide families and organisations with a tool to advocate for more funding.
The survey is anonymous and we will not ask for identifiable information. We will ask for your demographic and clinical details. We will also ask questions related to costs of health appointments, assessments, therapy, modifications, equipment, transport, respite, sport and leisure over the last 12 months, and over the last 5 years for major out of pocket costs related to living with cerebral palsy.
You do not have to complete the survey in one session. You can stop by clicking "save and return later" then return to it using the same link from the same computer/device. However the survey needs to be completed within one month.
You can withdraw from the survey at any time, without providing a reason, by exiting the browser window. If you exit the survey before it is finished, we may use the answers you have provided until that point. It will take between 30 to 90 minutes to complete the survey.
If you would like more information about the study, please contact Claire Galea -
[redacted]
If you are happy to take part in this survey, please tick Yes to "I have read and understood this information and I consent to my data being used for research and reporting purposes".
Your answers will only be accessible by the project team at the Research Institute Cerebral Palsy Alliance and will be kept on a secure server for 5 years.
This study was granted ethical approval by the Human Research Ethics Committee of the University of Sydney. If you have any complaints about this study please contact human.ethics@sydney.edu.au or call 02 8627 8176.
Thank you very much for taking the time to complete this survey and for sharing your information with us.
Claire, Sarah, Alison, Isabelle, Sophie, Fiona and James

[Attachment: "Participant Information Sheet.pdf"]

Participant Information Sheet

○ I have read and understood this information and I consent to my data being used for research and reporting purposes.

If these questions raise any serious concerns and you would like to talk to someone please contact your GP, your Health Professional or someone from the research team:
[redacted]

OR

HEADSPACE 1300 737 616 https://www.headspace.org.au/
LIFE LINE 13 11 14 (24hrs) https://www.lifeline.org.au/
BEYOND BLUE 1300 224 636
MindSpot 1800 614 434

2. Screening

Are you a person living with cerebral palsy?	◯ yes<br>◯ no
Are you living in Australia?	◯ yes<br>◯ no

Unfortunately the questions in this survey are not appropriate in your situation.
If you are caring for a person living with cerebral palsy please refer to this survey - insert link

We [redacted] would still like to share your experience, please email [redacted]

If you are interested in finding out more about this project, it will be completed in 2020. The Cerebral Palsy Alliance Research Institute will have more information available on their website when the project is finished.

3. Demographic section

What is your year of birth?

◯ 1935
◯ 1936
◯ 1937
◯ 1938
◯ 1939
◯ 1940
◯ 1941
◯ 1943
◯ 1944
◯ 1945
◯ 1946
◯ 1947
◯ 1948
◯ 1949
◯ 1950
◯ 1951
◯ 1952
◯ 1953
◯ 1954
◯ 1955
◯ 1956
◯ 1957
◯ 1958
◯ 1959
◯ 1960
◯ 1961
◯ 1962
◯ 1963
◯ 1964
◯ 1965
◯ 1966
◯ 1967
◯ 1968
◯ 1969
◯ 1970
◯ 1971
◯ 1972
◯ 1973
◯ 1974
◯ 1975
◯ 1976
◯ 1977
◯ 1978
◯ 1979
◯ 1980
◯ 1981
◯ 1982
◯ 1983
◯ 1984
◯ 1985
◯ 1986
◯ 1987
◯ 1988
◯ 1989
◯ 1990
◯ 1991
◯ 1992
◯ 1993
◯ 1994
◯ 1995
◯ 1996
◯ 1997
◯ 1998
◯ 1999
◯ 2000
◯ 2001
◯ 2002

Which gender do you identify with?	◯ female<br>◯ male<br>◯ gender neutral<br>◯ I don't wish to disclose
What is your highest completed level of education you achieved?	◯ primary education<br>◯ secondary education<br>◯ vocational training/diploma<br>◯ tertiary education<br>◯ none of the above
What are your current living arrangements?	◯ living with both parents<br>◯ living with one parent<br>◯ living with foster/adopted family<br>◯ living on my own independently<br>◯ living on my own with assistance<br>◯ living with a partner<br>◯ living with a partner and family<br>◯ living with friends<br>◯ living in a group home<br>◯ other
If other, please describe	__________________________________
What is your income (before tax)?	◯ less than $16,000 per year<br>◯ $15,600-$31,199 per year<br>◯ $31,200-$51,999 per year<br>◯ $52,000-$10,3999 per year<br>◯ $104,000-$259,999 per year<br>◯ $156,000-$259,999 per year<br>◯ $260,000 or more per year<br>◯ I do not wish to disclose<br>(Including your disability pension if you receive one)
Did you access disability funding in the last 12 months?	◯ Yes<br>◯ No<br>(E.g. NDIS, Better Start, other)
If yes, please specify which funding.	__________________________________
How much funding did you receive in the last 12 months? $	__________________________________<br>(number only)
What is your health insurance status?	☐ private<br>☐ bulk billed<br>☐ other<br>(Tick all that apply)

What is your main daytime occupation?	◯ education/school<br>◯ supported employment<br>◯ employed part time<br>◯ employed full time<br>◯ unemployed looking for a job<br>◯ unemployed not looking for a job (retired, housewife/husband, n/a)<br>◯ other
If other, please specify	__________________________________

4. Clinical details section

What is your predominant type of CP?

☐ Spastic (Muscles appear stiff and tight. From motor Cortex damage)
☐ Ataxic (Characterised by shaky movements. Affects balance and sense of positioning in space. From Cerebellum damage)
☐ Dyskinetic (Characterised by involuntary movements such as dystonia, athetosis and/or chorea. Damage to the Basal Ganglia)
☐ Hypotonic (Characterised by low muscle tone)
☐ I don't know
☐

Please choose the most appropriate level of severity:
For more information please refer to GMFCS (Gross Motor Functional Classification System) description form below

◯ Level I - I walk without limitations
◯ Level II - I walk with limitations
◯ Level III - I walk using a hand-held mobility device
◯ Level IV - I move around with limitations. I may use a powered mobility.
◯ Level V - I am transported in a wheelchair
◯ I don't know

GMFCS description form

[Attachment: "GMFCS-Adult-Graphics.jpg"]

Please tick for any other associated impairments and other conditions

☐ Epilepsy
☐ Intellectual
☐ Visual
☐ Hearing
☐ Speech
☐ ADHD
☐ ASD
☐ None
☐ Other
(Select as many as applicable)

If other, please describe

____

5. Health section

a) Medical expenses related to living with cerebral palsy

How many times did you see a GENERAL PRACTITIONER in the last 12 months?

- ◯ 0
- ◯ 1
- ◯ 2
- ◯ 3
- ◯ 4
- ◯ 5
- ◯ 6
- ◯ 7
- ◯ 8
- ◯ 9
- ◯ 10
- ◯ 11
- ◯ 12
- ◯ more than once a month

How was this funded?

- ☐ Community health - government funded
- ☐ Private health insurance
- ☐ Out of own pocket
- ☐ Charity/Non-profit organisation
- ☐ NDIS
- ☐ Better start
- ☐ Bulk billed / Medicare (incl. subsidised care plan)
- ☐ Employer paid
- ☐ Personal fundraising
- ☐ Other

(Select as many as applicable)

If other, please describe

____

Approximate out of pocket cost for you per visit?

____

(Enter number only i.e 56, if no out of pocket cost enter 0)

How many times did you see a NEUROLOGIST in the last 12 months?

- ◯ 0
- ◯ 1
- ◯ 2
- ◯ 3
- ◯ 4
- ◯ 5
- ◯ 6
- ◯ 7
- ◯ 8
- ◯ 9
- ◯ 10
- ◯ 11
- ◯ 12
- ◯ more than once a month

How was this funded?	☐ Community health - government funded<br>☐ Private health insurance<br>☐ Out of own pocket<br>☐ Charity/Non-profit organisation<br>☐ NDIS<br>☐ Better start<br>☐ Bulk billed / Medicare (incl. subsidised care plan)<br>☐ Employer paid<br>☐ Personal fundraising<br>☐ Other<br>(Select as many as applicable)
If other, please describe	__________________________________
Approximate out of pocket cost for you per visit?	__________________________________<br>(Enter number only i.e. 68, if no out of pocket cost enter 0)
How many times did you see a REHAB Specialist in the last 12 months?	◯ 0<br>◯ 1<br>◯ 2<br>◯ 3<br>◯ 4<br>◯ 5<br>◯ 6<br>◯ 7<br>◯ 8<br>◯ 9<br>◯ 10<br>◯ 11<br>◯ 12<br>◯ more than once a month
How was this funded?	☐ Community health - government funded<br>☐ Private health insurance<br>☐ Out of own pocket<br>☐ Charity/Non-profit organisation<br>☐ NDIS<br>☐ Better start<br>☐ Bulk billed / Medicare (incl. subsidised care plan)<br>☐ Employer paid<br>☐ Personal fundraising<br>☐ Other<br>(Select as many as applicable)
If other, please describe	__________________________________
Approximately out of pocket cost for you per visit?	__________________________________<br>(Enter number only, if no out of pocket cost enter 0)
Did you see any other specialist doctor(s) in the last 12 months?	◯ Yes<br>◯ No<br>(E.g. Cardiologist, Surgeon, Ear Nose and Throat Specialist)

If yes, which other specialist doctor(s) did you see in the last 12 months?	__________________________
How many times did you see this other specialist doctor(s) over the last 12 months?	◯ 1<br>◯ 2<br>◯ 3<br>◯ 4<br>◯ 5<br>◯ 6<br>◯ 7<br>◯ 8<br>◯ 9<br>◯ 10<br>◯ 11<br>◯ 12<br>◯ more than once a month
How was this funded?	☐ Community health - government funded<br>☐ Private health insurance<br>☐ Out of own pocket<br>☐ Charity/Non-profit organisation<br>☐ NDIS<br>☐ Better start<br>☐ Bulk billed / Medicare (incl. subsidised care plan)<br>☐ Employer paid<br>☐ Personal fundraising<br>☐ Other<br>(Select as many as applicable)
If other, please describe	__________________________
Approximately out of pocket cost for you per visit?	__________________________<br>(Enter number only i.e. 57, if no out of pocket cost enter 0)

5. Health section

b) Allied Health Practitioners expenses related to living with cerebral palsy

How many times did you see a Allied Health Practitioner in the last 12 months?

		once a week	once a fortnight	once a month	once every 3 months	once every 6 months	once every 12 months	not applicable / never
1	Physiotherapist	◯	◯	◯	◯	◯	◯	◯
2	Speech Therapist	◯	◯	◯	◯	◯	◯	◯
3	Occupational Therapist	◯	◯	◯	◯	◯	◯	◯

How was this funded?

- ☐ Community health - government funded
- ☐ Private health insurance
- ☐ Out of own pocket
- ☐ Charity/Non-profit organisation
- ☐ NDIS
- ☐ Better start
- ☐ Bulk billed / Medicare (incl. subsidised care plan)
- ☐ Employer paid
- ☐ Personal fundraising
- ☐ Other

(Select as many as applicable)

If other, please describe

____

Approximate out of pocket costs for Allied Health therapy per visit?

____

(Enter number only i.e 45, if no out of pocket cost enter 0)

Approximate out of pocket costs for Allied Health assessments and reports over the last 12 months?

____

(If no cost, enter 0)

Did you see any other Allied Health Practitioner in the last 12 months?

◯ Yes
◯ No
(E.g. Social worker, Psychologist)

Please list type of Allied Health practitioner

____

How many times did you see this other Allied Health Practitioner in the last 12 months?

- ◯ 1
- ◯ 2
- ◯ 3
- ◯ 4
- ◯ 5
- ◯ 6
- ◯ 7
- ◯ 8
- ◯ 9
- ◯ 10
- ◯ 11
- ◯ 12
- ◯ more than once a month

(E.g. Social worker, Psychologist)

How was this funded?

- ☐ Community health - government funded
- ☐ Private health insurance
- ☐ Out of own pocket
- ☐ Charity/Non-profit organisation
- ☐ NDIS
- ☐ Better start
- ☐ Bulk billed / Medicare (incl. subsidised care plan)
- ☐ Employer paid
- ☐ Personal fundraising
- ☐ Other

(Select as many as applicable)

If other, please describe

____

Approximate out of pocket costs for other Allied Health therapy per visit?

____

(Enter number only, if no out of pocket cost enter 0)

Have you had alternative treatments?

- ◯ Yes
- ◯ No

(E.g. acupuncture, massages,...)

Please list type of alternative treatment(s)

____

How was this funded?

- ☐ Community health - government funded
- ☐ Private health insurance
- ☐ Out of own pocket
- ☐ Charity/Non-profit organisation
- ☐ NDIS
- ☐ Better start
- ☐ Bulk billed / Medicare (incl. subsidised care plan)
- ☐ Employer paid
- ☐ Personal fundraising
- ☐ Other

(Select as many as applicable)

If other, please describe

____

If yes, approximate out of pocket costs for alternative treatment(s) per visit?

____
(Enter number only, if no out of pocket cost enter 0)

5. Health section

c) Medication expenses related to living with cerebral palsy

Have you purchased any medications in the past 12 months?	◯ Yes<br>◯ No
How was this funded?	☐ Community health - government funded<br>☐ Private health insurance<br>☐ Out of own pocket<br>☐ Charity/Non-profit organisation<br>☐ NDIS<br>☐ Better start<br>☐ Bulk billed / Medicare (incl. subsidised care plan)<br>☐ Employer paid<br>☐ Personal fundraising<br>☐ Other<br>(Select as many as applicable)
If other, please describe	______________________________
Out of pocket costs for medications over the last 12 months for tone management, pain, constipation, epilepsy or re-flux, mental health.	______________________________<br>(Enter number only i.e. 125)

5. Health section

d) Hospitalisation(s) related to living with cerebral palsy

30%
.

How many times were you hospitalised in the last 12 months?	◯ 0<br>◯ 1<br>◯ 2<br>◯ 3<br>◯ 4<br>◯ 5<br>◯ 6<br>◯ 7<br>◯ 8<br>◯ 9<br>◯ 10<br>◯ 11<br>◯ 12<br>◯ more than once a month
Main reason for admission(s):	__________________________________
How was this funded?	☐ Community health - government funded<br>☐ Private health insurance<br>☐ Out of own pocket<br>☐ Charity/Non-profit organisation<br>☐ NDIS<br>☐ Better start<br>☐ Bulk billed / Medicare (incl. subsidised care plan)<br>☐ Employer paid<br>☐ Personal fundraising<br>☐ Other<br>(Select as many as applicable)
If other, please describe	__________________________________
Approximate out of pocket costs for hospitalisation over the last 12 months?	__________________________________<br>(If no cost, enter 0)
Thinking about all these healthcare costs (GP visits, specialist doctors, allied health, medication and hospitalisations), would you have spent more on your health if you could afford it?	◯ Yes<br>◯ No
If yes, how much approximately over the last 12 months?	◯ less than $2000<br>◯ between $2000 and $5000<br>◯ more than $5000
Please specify what you would have spent more on:	__________________________________<br>(E.g. elective surgery, medications, physiotherapy, ...)

Is there anything else you would like to share about Healthcare costs?

____

6. Daily living section

a) Communication expenses related to living with cerebral palsy.

Question	Response
Have you purchased specialised equipment to communicate over the last 5 years?	◯ Yes<br>◯ No<br>(E.g. speech generating devices, symbol boards, ...)
If yes, please specify	__________________________________
How was this funded?	☐ Community health - government funded<br>☐ Private health insurance<br>☐ Out of own pocket<br>☐ Charity/Non-profit organisation<br>☐ NDIS<br>☐ Better start<br>☐ Bulk billed / Medicare (incl. subsidised care plan)<br>☐ Employer paid<br>☐ Personal fundraising<br>☐ Other<br>(Select as many as applicable)
If other, please describe	__________________________________
Approximate out of pocket costs for you over the last 5 years for communication?	__________________________________<br>(Enter number only, if no out of pocket cost enter 0)

6. Daily living section

b) Mobility expenses related to living with cerebral palsy

Have you purchased specialised equipment for mobility over the last 5 years?

◯ Yes
◯ No
(E.g. mobility scooter, specialised seats, walking device, standing frame, lower limb orthoses)

If yes, please specify

____

How was this funded?

☐ Community health - government funded
☐ Private health insurance
☐ Out of own pocket
☐ Charity/Non-profit organisation
☐ NDIS
☐ Better start
☐ Bulk billed / Medicare (incl. subsidised care plan)
☐ Employer paid
☐ Personal fundraising
☐ Other
(Select as many as applicable)

If other, please describe

____

Approximate out of pocket cost for you over the last 5 years for mobility?

____
(Enter number only, if no out of pocket cost enter 0)

6. Daily living section
c) Nutrition/Clothing/Personal care expenses related to living with cerebral palsy

Have you had any extra costs related to nutrition?
- ◯ Yes
- ◯ No

(E.g. special meals, supplements, thickeners, food utensils)

If yes, please specify

____

How was this funded?
- ☐ Community health - government funded
- ☐ Private health insurance
- ☐ Out of own pocket
- ☐ Charity/Non-profit organisation
- ☐ NDIS
- ☐ Better start
- ☐ Bulk billed / Medicare (incl. subsidised care plan)
- ☐ Employer paid
- ☐ Personal fundraising
- ☐ Other

(Select as many as applicable)

If other, please describe

____

Approximate out of pocket amount for you over the last 12 months for nutrition?

____
(Enter number only, if no out of pocket cost enter 0)

Have you had any extra costs related to clothing?
- ◯ Yes
- ◯ No

(E.g. adapted clothing, alterations, shoes)

If yes, please specify

____

How was this funded?
- ☐ Community health - government funded
- ☐ Private health insurance
- ☐ Out of own pocket
- ☐ Charity/Non-profit organisation
- ☐ NDIS
- ☐ Better start
- ☐ Bulk billed / Medicare (incl. subsidised care plan)
- ☐ Employer paid
- ☐ Personal fundraising
- ☐ Other

(Select as many as applicable)

If other, please describe

____

Approximate out of pocket cost for you over the last 12 months for clothing?

____
(Enter number only, if no out of pocket cost enter 0)

Have you had any extra costs related to personal care items?	◯ Yes<br>◯ No<br>(E.g. incontinence pads, bandages, special toothbrush)
If yes, please specify	______________________________
How was this funded?	☐ Community health - government funded<br>☐ Private health insurance<br>☐ Out of own pocket<br>☐ Charity/Non-profit organisation<br>☐ NDIS<br>☐ Better start<br>☐ Bulk billed / Medicare (incl. subsidised care plan)<br>☐ Employer paid<br>☐ Personal fundraising<br>☐ Other<br>(Select as many as applicable)
If other, please describe	______________________________
Approximate out of pocket cost for you over the last 12 months for personal care?	______________________________<br>(Enter number only i.e. 58, if no out of pocket cost enter 0)
Would you have spent more on assistive technology (for communication and mobility) or equipment for daily living if you could afford it?	◯ Yes<br>◯ No
If yes, how much approximately over the last 12 months?	◯ more than $2000<br>◯ more than $5000<br>◯ more than $10 000
Please specify:	______________________________________
Is there anything else you would like to share about your Daily Living costs?	______________________________________<br>(E.g. mobility, equipment, personal care,...)

50%
.

7. House section

a) Modification(s) expenses related to living with cerebral palsy

Have you had to move to another location for residential housing related to your condition?	◯ Yes<br>◯ No
If yes, please describe:	__________________________
If yes, what were the out of pocket costs associated with this change of location?	______________________<br>(Enter number only, if no out of pocket cost enter 0)
Have you changed the access to / into your home?	◯ Yes<br>◯ No<br>(E.g. driveway, access to garden, access to balcony, parking, ramps)
How was this funded?	☐ Community health - government funded<br>☐ Private health insurance<br>☐ Out of own pocket<br>☐ Charity/Non-profit organisation<br>☐ NDIS<br>☐ Better start<br>☐ Bulk billed / Medicare (incl. subsidised care plan)<br>☐ Employer paid<br>☐ Personal fundraising<br>☐ Other<br>(Select as many as applicable)
If other, please describe	______________________
What was the out of pocket amount for you over the last 5 years for these home access modifications?	______________________<br>(Enter number only, if no out of pocket cost enter 0)
Have you had modifications done inside your house?	◯ Yes<br>◯ No<br>(E.g. bathroom, living room, bedroom)
How was this funded?	☐ Community health - government funded<br>☐ Private health insurance<br>☐ Out of own pocket<br>☐ Charity/Non-profit organisation<br>☐ NDIS<br>☐ Better start<br>☐ Bulk billed / Medicare (incl. subsidised care plan)<br>☐ Employer paid<br>☐ Personal fundraising<br>☐ Other<br>(Select as many as applicable)

If other, please describe	__________________________________
What was the out of pocket amount for you over the last 5 years for these inside home modifications?	__________________________________<br>(Enter number only, if no out of pocket cost enter 0)

7. House section

b) Equipment expenses related to living with cerebral palsy

Have you purchased specific equipment for your house related to having cerebral palsy?

- ◯ Yes
- ◯ No

(E.g. mattress, hoist, sling, shower chair, alarm bell, special bath)

If yes, please describe

____

How was this funded?

- ☐ Community health - government funded
- ☐ Private health insurance
- ☐ Out of own pocket
- ☐ Charity/Non-profit organisation
- ☐ NDIS
- ☐ Better start
- ☐ Bulk billed / Medicare (incl. subsidised care plan)
- ☐ Employer paid
- ☐ Personal fundraising
- ☐ Other

(Select as many as applicable)

If other, please describe

____

What was the out of pocket amount over the last 5 years?

____

(Enter number only, if no out of pocket cost enter 0)

Would you have spent more on your home environment if you could afford it?

- ◯ Yes
- ◯ No

Please specify:

____

If yes, how much approximately over the last 5 years?

- ◯ less than $2000
- ◯ between $2000 and $5000
- ◯ more than $5000

8. Transport section
a) Car expenses related to living with cerebral palsy

Have you purchased a specific type or model of car to accommodate your specific needs?

◯ Yes
◯ No

If yes, please specify

____
(E.g. automatic vs manual, bigger type, different model)

How was this funded?

☐ Community health - government funded
☐ Private health insurance
☐ Out of own pocket
☐ Charity/Non-profit organisation
☐ NDIS
☐ Better start
☐ Bulk billed / Medicare (incl. subsidised care plan)
☐ Employer paid
☐ Personal fundraising
☐ Other
(Select as many as applicable)

If other, please describe

____

What was the estimated price difference / out of pocket amount for you over the last 5 years?

____
(Enter number only, if no out of pocket cost enter 0)

8. Transport section
b) Car modification(s) expenses related to living with cerebral palsy

Have you had modifications done to your car to accommodate your specific needs?	◯ Yes<br>◯ No<br>(E.g. ramp for wheelchair, hydraulic ramp, steering wheel, handbrake, automatic gears, modification to the seat, or interior)
If yes, please specify modifications:	__________________________________
How was this funded?	☐ Community health - government funded<br>☐ Private health insurance<br>☐ Out of own pocket<br>☐ Charity/Non-profit organisation<br>☐ NDIS<br>☐ Better start<br>☐ Bulk billed / Medicare (incl. subsidised care plan)<br>☐ Employer paid<br>☐ Personal fundraising<br>☐ Other<br>(Select as many as applicable)
If other, please describe	__________________________________
What was the out of pocket amount for you over the last 5 years for these modifications?	__________________________________<br>(Enter number only, if no out of pocket cost enter 0)
Have you had other out of pocket costs associated with car requirements?	◯ Yes<br>◯ No
If yes, please describe	__________________________________
What was the out of pocket amount for you over the last 5 years?	__________________________________<br>(Enter number only, if no out of pocket cost enter 0)

65%
.

8. Transport section

c) Other transport expenses related to living with cerebral palsy

Have you used public transport to attend health related appointments?

◯ Yes
◯ No

How was this funded?

☐ Community health - government funded
☐ Private health insurance
☐ Out of own pocket
☐ Charity/Non-profit organisation
☐ NDIS
☐ Better start
☐ Bulk billed / Medicare (incl. subsidised care plan)
☐ Employer paid
☐ Personal fundraising
☐ Other
(Select as many as applicable)

If other, please describe

____

What was the out of pocket amount for you over the last 12 months ?

____
(Enter number only, if no out of pocket cost enter 0)

Have you paid for parking and/or tolls to attend health related appointments over the last 12 months?

◯ Yes
◯ No

How was this funded?

☐ Community health - government funded
☐ Private health insurance
☐ Out of own pocket
☐ Charity/Non-profit organisation
☐ NDIS
☐ Better start
☐ Bulk billed / Medicare (incl. subsidised care plan)
☐ Employer paid
☐ Personal fundraising
☐ Other
(Select as many as applicable)

If other, please describe

____

What was the out of pocket amount for you over the last 12 months ?

____
(Enter number only, if no out of pocket cost enter 0)

Have you paid for other means of transportation to attend health related appointments over the last 12 months?

◯ Yes
◯ No
(E.g. Uber, Taxi,...)

How was this funded?	☐ Community health - government funded<br>☐ Private health insurance<br>☐ Out of own pocket<br>☐ Charity/Non-profit organisation<br>☐ NDIS<br>☐ Better start<br>☐ Bulk billed / Medicare (incl. subsidised care plan)<br>☐ Employer paid<br>☐ Personal fundraising<br>☐ Other<br>(Select as many as applicable)
If other, please describe	__________________________________
What was the out of pocket amount for you over the last 12 months ?	__________________________________<br>(Enter number only, if no out of pocket cost enter 0)
Is there anything else you would like to share regarding your transport costs?	____

9. Employment section
Expenses related to living with cerebral palsy

If you are unemployed, are there any out of pocket costs related to your day time occupation? If yes, please specify.

____
(E.g. training, mealtime help, ...)

Did you need employment support?

◯ Yes
◯ No
(E.g. readiness program, training)

If yes, please specify

____

How was this funded?

☐ Community health - government funded
☐ Private health insurance
☐ Out of own pocket
☐ Charity/Non-profit organisation
☐ NDIS
☐ Better start
☐ Bulk billed / Medicare (incl. subsidised care plan)
☐ Employer paid
☐ Personal fundraising
☐ Other
(Select as many as applicable)

If other, please describe

____

What was the out of pocket amount for you over the last 12 months ?

____
(Enter number only i.e. 56, if no out of pocket cost enter 0)

Did you need access modifications or specific equipment at your place of employment?

◯ Yes
◯ No
(E.g. ramps, facilities)

If yes, please specify

____

How was this funded?

☐ Community health - government funded
☐ Private health insurance
☐ Out of own pocket
☐ Charity/Non-profit organisation
☐ NDIS
☐ Better start
☐ Bulk billed / Medicare (incl. subsidised care plan)
☐ Employer paid
☐ Personal fundraising
☐ Other
(Select as many as applicable)

If other, please describe

____

What was the out of pocket amount for you over the last 12 months ?	__________________________________ (Enter number only, if no out of pocket cost enter 0)
Have you had any other additional costs related to your employment?	◯ Yes<br>◯ No<br>( )
If yes, please specify	__________________________________
How was this funded?	☐ Community health - government funded<br>☐ Private health insurance<br>☐ Out of own pocket<br>☐ Charity/Non-profit organisation<br>☐ NDIS<br>☐ Better start<br>☐ Bulk billed / Medicare (incl. subsidised care plan)<br>☐ Employer paid<br>☐ Personal fundraising<br>☐ Other<br>(Select as many as applicable)
If other, please describe	__________________________________
Approximate out of pocket cost for you over the last 5 years?	__________________________________ (Enter number only, if no out of pocket cost enter 0)
Would you have spent more on employment (or getting ready to be employed) if you could afford it?	◯ Yes<br>◯ No
If yes, please specify	____
How much approximately over the last 5 years?	◯ less than $2000<br>◯ between $2000 and $5000<br>◯ more than $5000

80%
.

10. Respite section

Expenses related to living with cerebral palsy

Have you had respite care in the last 12 months?	◯ Yes<br>◯ No<br>(E.g. day setting, overnight respite)
If yes, how often?	______________________
If yes, please specify	______________________
How was this funded?	☐ Community health - government funded<br>☐ Private health insurance<br>☐ Out of own pocket<br>☐ Charity/Non-profit organisation<br>☐ NDIS<br>☐ Better start<br>☐ Bulk billed / Medicare (incl. subsidised care plan)<br>☐ Employer paid<br>☐ Personal fundraising<br>☐ Other<br>(Select as many as applicable)
If other, please describe	______________________
Approximate out of pocket cost for you over the last 12 months?	______________________<br>(Enter number only, if no out of pocket cost enter 0)
Would you have spent more on respite if you could afford it?	◯ Yes<br>◯ No
If yes, please specify	___________________________
How much approximately in the last 5 years?	◯ less than $2000<br>◯ between $2000 and $5000<br>◯ more than $5000

11. Sport and Leisure section

Expenses related to living with cerebral palsy

Have you been on holiday(s) that accommodated and catered to your specific needs over the last 12 months?	◯ Yes<br>◯ No
If yes, please specify	______________________________<br>(E.g. cruise ship with accessible cabin)
How was this funded?	☐ Community health - government funded<br>☐ Private health insurance<br>☐ Out of own pocket<br>☐ Charity/Non-profit organisation<br>☐ NDIS<br>☐ Better start<br>☐ Bulk billed / Medicare (incl. subsidised care plan)<br>☐ Employer paid<br>☐ Personal fundraising<br>☐ Other<br>(Select as many as applicable)
If other, please describe	______________________________
If no, what were the reasons?	______________________________<br>(E.g. no money for carer, no space in the car, availability of accessible room)
What was the approximate 'excess' amount spent over the last 12 months for your holidays?	______________________________<br>(Enter number only, excess = on top of usual cost)
Would you have spent money on holidays if you could afford it?	◯ Yes<br>◯ No
If yes, please specify	______________________________
If yes, how much approximately in the last 12 months?	◯ less than $2000<br>◯ between $2000 and $5000<br>◯ more than $5000<br>(Enter number only)
Is there anything else you would like to say about the cost of going on holidays?	______________________________
Have you attended sports or leisure activities that catered to your specific needs?	◯ Yes<br>◯ No

If yes, please specify

____

How was this funded?

☐ Community health - government funded
☐ Private health insurance
☐ Out of own pocket
☐ Charity/Non-profit organisation
☐ NDIS
☐ Better start
☐ Bulk billed / Medicare (incl. subsidised care plan)
☐ Employer paid
☐ Personal fundraising
☐ Other
(Select as many as applicable)

If other, please describe

____

What was the 'excess' amount spent over the last 12 months on sports leisure activities?

____
(Enter number only, excess = on top of usual cost)

If no, what were the reasons?

____
(E.g. not adapted to my specific needs, too far, too costly,...)

Is there anything else you would like to say about the cost of participating in sport or leisure activities?

____

Have you had other out of pocket costs associated with playing a sport or participating in leisure activities?

◯ Yes
◯ No
(E.g. modifications to equipment, extra insurance)

If yes, please specify

____

Approximate out of pocket cost for you over the last 12 months?

____
(Enter number only)

Would you have spent more on holidays, sport and leisure activities if you could afford it?

◯ Yes
◯ No

If yes, please specify

____

How much approximately in the last 12 months?

◯ less than $2000
◯ between $2000 and $5000
◯ more than $5000

95%

.

12. Financial distress (InCharge Financial distress/Financial Well-being-Scale)

What do you feel is the level of your financial stress today?
Overwhelming stress High stress Low stress No stress at all 1 2 3 4 5 6 7 8 9 10

____
(enter a number from 1-10)

How satisfied are you with your present financial situation?
Dissatisfied Satisfied 1 2 3 4 5 6 7 8 9 10

____
(enter a number from 1-10)

How do you feel about your current financial situation?
Feel overwhelmed Sometimes feel worried Rarely worry Never worry 1 2 3 4 5 6 7 8 9 10

____
(enter a number from 1-10)

How often do you worry about being able to meet normal monthly living expenses?
Worry all the time Sometimes worry Rarely worry Never worry
1 2 3 4 5 6 7 8 9 10

____
(enter a number from 1-10)

How confident are you that you could find the money to pay for a financial emergency that costs about $1000?
No confidence Little confidence Some confidence
High confidence 1 2 3 4 5 6 7 8 9 10

____
(enter a number from 1-10)

How often does this happen to you? you want to go out to eat, go to a movie or do something else and don't go because you can't afford to?
All the time Sometimes Rarely Never 1 2 3 4 5 6 7 8 9 10

____
(enter a number from 1 -10)

How frequently do you find yourself just getting by financially and living pay check to pay check?
All the time Sometimes Rarely Never 1 2 3 4 5 6 7 8 9 10

____
(enter a number from 1 -10)

How stressed do you feel about your personal finances in general?
Overwhelming stress High stress Low stress No stress at all 1 2 3 4 5 6 7 8 9 10

____
(enter a number from 1 - 10)

Thank you!

Is there anything else you want to tell us about the financial cost, or the level of financial stress, of living with cerebral palsy ?

____

Thank you for taking the time to help us better understand the financial costs of living with cerebral palsy!

- ☐ I would like to be kept up to date with the results of this study
- ☐ I can be contacted for more details if required at a later stage of this study
- ☐ I would like to receive more invitations to participate in CP research studies and I would like to know more about the CP Register
- ☐ I would like to be involved in CP research and I would like to know more about CP Quest
- ☐ I would like to have a chance to win a $50 voucher as a token of appreciation for completing this survey

(Select as many as applicable)

Please provide your email address here if you ticked one or more options. Your email address will not be exported for data-analysis.

If you do not wish to provide an email address through this survey please contact the researchers directly - [redacted]

____

100%

.
